# Human Rap1 modulates TRF2 attraction to telomeric DNA

**DOI:** 10.1093/nar/gkv097

**Published:** 2015-02-11

**Authors:** Eliška Janoušková, Ivona Nečasová, Jana Pavloušková, Michal Zimmermann, Milan Hluchý, Victoria Marini, Monika Nováková, Ctirad Hofr

**Affiliations:** 1 Chromatin Molecular Complexes, CEITEC and Laboratory of Functional Genomics and Proteomics, National Centre for Biomolecular Research, Faculty of Science, Masaryk University, Brno CZ-62500, Czech Republic; 2 Department of Biology, Faculty of Medicine, Masaryk University, Brno CZ-62500, Czech Republic

## Abstract

More than two decades of genetic research have identified and assigned main biological functions of shelterin proteins that safeguard telomeres. However, a molecular mechanism of how each protein subunit contributes to the protecting function of the whole shelterin complex remains elusive. Human Repressor activator protein 1 (Rap1) forms a multifunctional complex with Telomeric Repeat binding Factor 2 (TRF2). Rap1–TRF2 complex is a critical part of shelterin as it suppresses homology-directed repair in Ku 70/80 heterodimer absence. To understand how Rap1 affects key functions of TRF2, we investigated full-length Rap1 binding to TRF2 and Rap1–TRF2 complex interactions with double-stranded DNA by quantitative biochemical approaches. We observed that Rap1 reduces the overall DNA duplex binding affinity of TRF2 but increases the selectivity of TRF2 to telomeric DNA. Additionally, we observed that Rap1 induces a partial release of TRF2 from DNA duplex. The improved TRF2 selectivity to telomeric DNA is caused by less pronounced electrostatic attractions between TRF2 and DNA in Rap1 presence. Thus, Rap1 prompts more accurate and selective TRF2 recognition of telomeric DNA and TRF2 localization on single/double-strand DNA junctions. These quantitative functional studies contribute to the understanding of the selective recognition of telomeric DNA by the whole shelterin complex.

## INTRODUCTION

Telomeres are essential nucleoprotein structures located at the ends of linear chromosomes. The main function of telomeres is to protect the very ends of chromosomal DNA from nucleolytic degradation, unwanted DNA repair processes and fatal chromosome fusions. Furthermore, telomeres shorten due to the incomplete DNA replication in each cell cycle. Hence, telomeres are closely connected with the molecular mechanisms of cell aging. The shortening of telomeres can be reverted by the telomerase, which actively extends telomeric DNA by adding oligonucleotide repeats to chromosomal ends. Telomerase access to telomeric DNA as well as the inhibition of DNA damage response and repair pathways at telomeres is controlled by proteins that selectively bind telomeric DNA. In mammalian cells, six telomeric proteins form a functional complex called shelterin (1). Shelterin protects telomeres from being recognized as double-strand breaks and subsequent activation of DNA damage signaling and repair pathways (2). A central role in shelterin regarding its protective functions is played by Telomeric Repeat binding Factor 2 (TRF2). TRF2 specifically inhibits Ataxia Telangiectasia Mutated (ATM) kinase-dependent DNA damage signaling and the classical Ku70/80- and Ligase IV-mediated non-homologous end-joining pathway at telomeres (3–5). The molecular mechanism of TRF2 action has not been fully elucidated. One possible explanation surfaced from the finding that TRF2 alters the spatial arrangement of telomeric DNA by formation of specific telomeric loop structures (t-loops) (6,7). T-loops physically hide DNA ends from DNA damage sensors, DNA repair enzymes and telomerase (8). Moreover, TRF2 prevents the resolution of the t-loop structure by repair enzymes (9). *In vivo*, TRF2 forms a stable complex with Repressor activator protein 1 (Rap1) (10). The Rap1–TRF2 complex was shown to effectively suppress homology-directed repair of chromosome ends in the absence of Ku 70/80 (11). While TRF2 is life essential (12), Rap1 deletion does not affect cell viability (11) nor telomere protection *in vivo*, as has been shown recently (13). All these findings provoke questions regarding real functions of Rap1–TRF2 complex as a part of telomere maintenance machinery.

**Figure 1.**
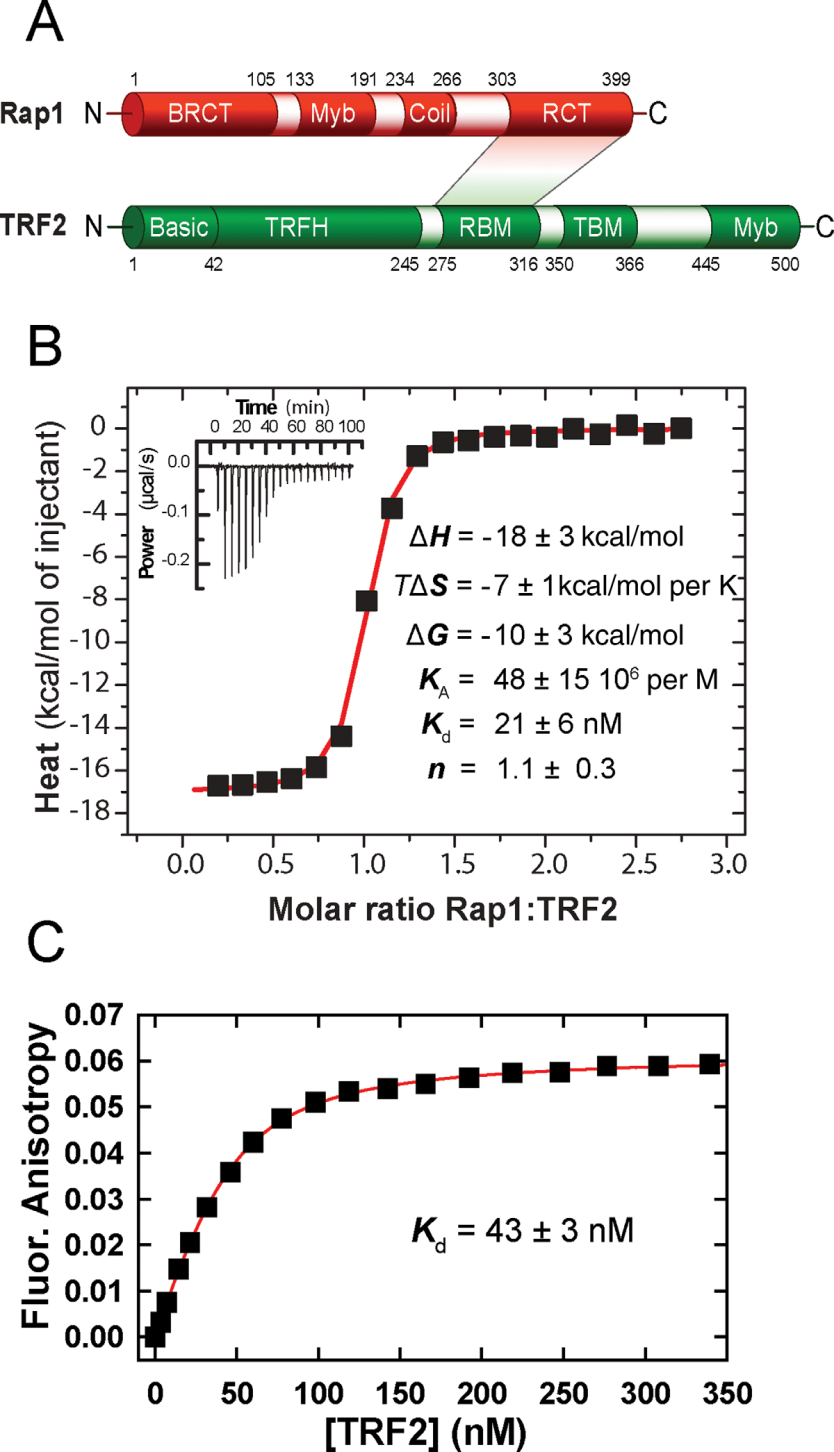
Structure, interaction regions and binding of full-length proteins Rap1 and TRF2. (**A**) Rap1 and TRF2 domain structure. In Rap1, N-terminal BRCT domain; Myb domain; coiled-coil region, C-terminal specific protein interaction RCT domain. In TRF2, N-terminal basic domain, C-terminal DNA-binding Myb domain, TRFH dimerization domain; RBM–Rap1 binding motif, TIN2-binding motif TBM. The shaded area denominates directly interacting regions. (**B**) Isothermal titration calorimetry of Rap1 (44 μM) binding to TRF2 (5 μM) in 50 mM NaCl and 50 mM sodium phosphate buffer (pH 7.0) at 25°C. The control ITC titration of Rap1 into the cell containing buffer has been used for a proper data normalization and baseline subtraction. The inset represents 20 injections of 10 μl of Rap1 into a reaction cell containing TRF2. (**C**) Binding affinity of TRF2 to fluorescently labeled Rap1. TRF2 was allowed to bind with Rap1 labeled by AlexaFluor 594 (100 nM). The dissociation constant was determined from non-linear fitting of the binding data (shown in red).

Human TRF2 consists of a basic N-terminal domain, a TRF homology (TRFH) domain mediating homodimerization of TRF2, a flexible linker region comprising Rap1 binding motif and a C-terminal DNA Myb domain (14) (Figure 1A). The myeloblastosis family of transcription factors DNA binding domain (Myb) of TRF2 binds the duplex DNA sequence 5′YTAGGGTTR, showing a subtle tolerance for canonical single-base exchanges (14,15). Human Rap1 comprises four protein interaction domains, pointing to the multifunctional character of the protein (11). The N-terminal part of Rap1 accommodates a domain of a breast cancer susceptibility protein that appears on its C-terminus (BRCT), the central part features a structural region showing Myb domain homology and a coiled-coil domain and the C-terminal part accommodates the Rap1-specific protein-interaction domain (RCT domain) (11) (Figure 1A). The RCT domain of Rap1 is critical for its interaction with TRF2 (16). The structural data of isolated TRF2 and Rap1 binding motifs reveal that driving protein–protein interactions are mediated mainly through hydrophobic amino acids (17). Even though live cell studies show a functional significance of TRF2–Rap1 interaction, the studies characterizing direct interactions of the two full-length proteins have been limited (17,18). Furthermore, even though it has been shown that Rap1 can affect binding of TRF2 to telomeric DNA *in vitro* (18), no significant effects of Rap1 on TRF2 binding to telomeres were observed in cells (13,19). The possible direct impact of Rap1 on the DNA-binding activity of TRF2 has not been described in a fully quantitative manner using equilibrium techniques. In order to address the effect of Rap1 on TRF2 binding properties, we performed extensive measurements of Rap1–TRF2–DNA interactions using a combination of quantitative methods, such as fluorescence anisotropy (FA), isothermal titration calorimetry (ITC), gel retardation analysis and surface plasmon resonance (SPR). We quantify how human Rap1 contributes to DNA-binding selectivity of TRF2. For the first time we describe that Rap1 induces a partial release of TRF2 from double-stranded telomeric DNA. Additionally, we propose a molecular mechanism of the Rap1–TRF2 preference for the telomeric DNA sequence.

## MATERIALS AND METHODS

### Cloning, expression and purification of proteins

The cDNA sequences of Rap1 and TRF2 were synthesized by Source BioScience and cloned to pDONR/Zeo vector (Life Technologies) using two sets of primers (Supplementary Table S1) and BP clonase enzyme mix from Gateway technology (Life Technologies). Resulting plasmid pDONR/Zeo *rap1/trf2* was cloned into pHGWA vector (20) using LR clonase enzyme mix (Life Technologies).

Recombinant TRF2 and Rap1 with N-terminal His_6_-tags were expressed in bacterial cells as described elsewhere (21,22). Briefly, TRF2 was expressed in *Escherichia coli* BL21(DE3) and Rap1 in *E. coli* BL21(DE3)RIPL carrying the vector pHGWA (20). After induction of proteins expression with 1mM isopropyl β-D-thiogalactoside, cells were harvested and resuspended in lysis buffer (50 mM sodium phosphate, 500 mM NaCl, 10 mM imidazole, 10% glycerol and 0.5% Tween 20, pH 8.0) containing protease inhibitors Leupeptin (4 μM) and Pepstatin (5 μM). Sonicated cell extracts were cleared by centrifugation and subsequent filtration (0.45 μm SterivexTM filter, Millipore). Supernatant containing protein TRF2 or Rap1 was further purified by Immobilized Metal ion Affinity Chromatography using TALON Metal Affinity Resin (Clontech) containing Co^2+^ cations as described (23). The proteins were eluted at 300 mM imidazole. The fractions containing pure protein were dialyzed into buffer composed of 50 mM sodium phosphate with 50 mM NaCl (pH 7.0) and subsequently, proteins were concentrated by ultrafiltration (Amicon 30K, Millipore).

The concentration of purified proteins was determined using the Bradford assay and their purity was verified by electrophoresis in sodium dodecyl sulfate (SDS)-polyacrylamide gel which was subsequently stained using Bio-Safe Coomassie G 250 (Bio-Rad). Mass spectrometry measurements were used to confirm that proteins were expressed in full length and high purity. Supplementary Figure S1 shows SDS-polyacrylamide gel electrophoresis of TRF2 and Rap1 used in the studies.

### DNA substrates

**Figure 2.**
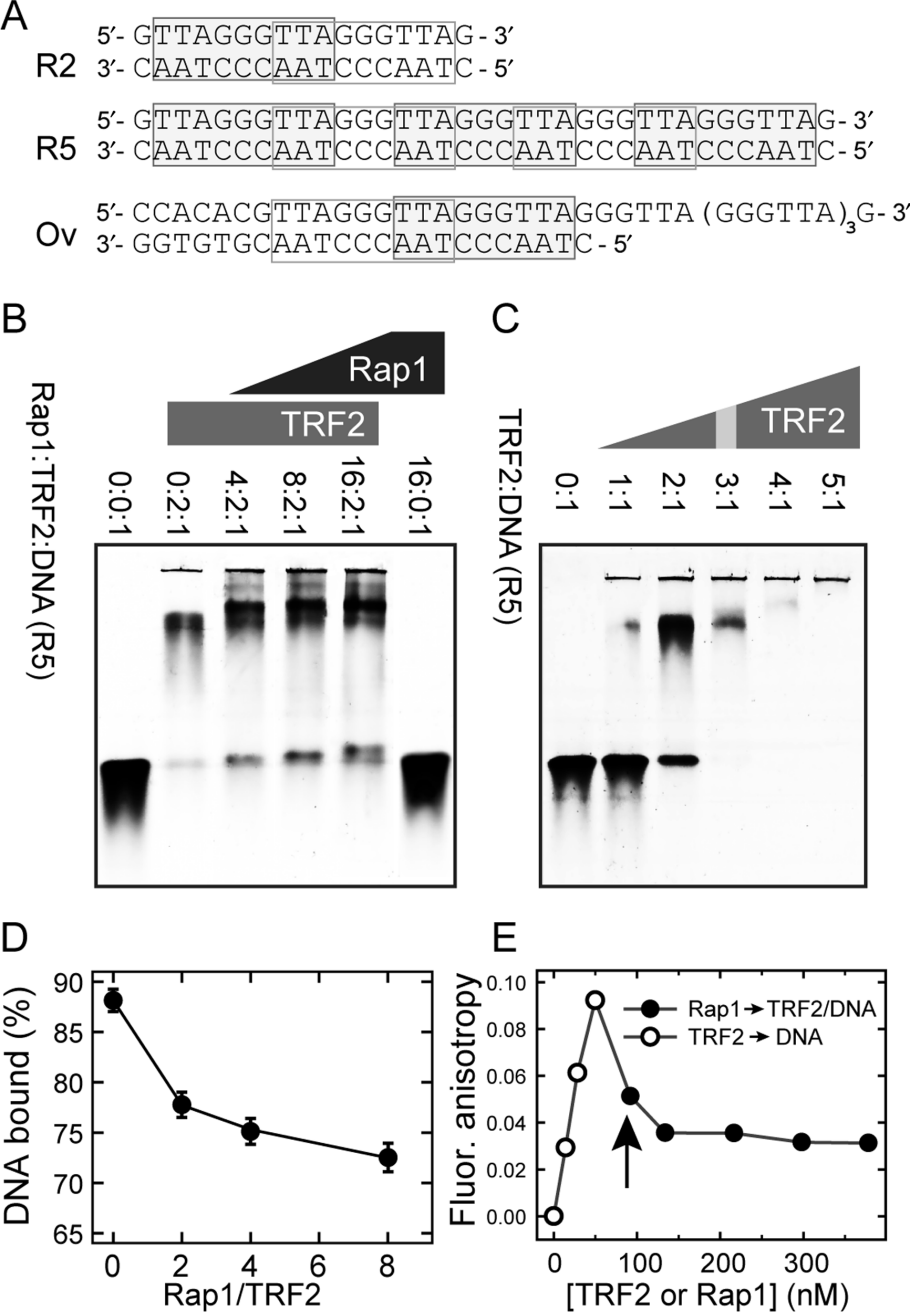
Protein Rap1 induces TRF2 release from telomeric DNA. (**A**) Sequences of telomeric DNA duplexes R2, R5 and Ov. Putative binding sites of TRF2 Myb domain are denoted by rectangles (13). (**B**) The intensity increase of the band corresponding to free DNA after addition of Rap1 monitored by EMSA. DNA oligonucleotide duplex R5 (5 pmol) labeled with Alexa Fluor 488 was incubated with constant amount of TRF2 (10 pmol) and increasing amount of Rap1 (20–80 pmol). The numbers above electrophoretic lanes represent the molar ratios of Rap1:TRF2:DNA in individual wells. Each ratio of Rap1 was prepared in triplicates to improve the accuracy of free DNA quantification. Reaction mixtures were resolved on horizontal 5% non-denaturing polyacrylamide gel. (**C**) Saturated TRF2 binding to telomeric DNA duplex. Reaction mixtures (15 μl) contained the same amount of fluorescently labeled DNA duplex R5 (3 pmol) and increasing amounts of TRF2. Numbers above electrophoretic lanes represent the molar ratio of TRF2:DNA. The ratio corresponding to the binding saturation is indicated with the gray stripe. (**D**) The quantification of DNA bound to TRF2 in the presence of Rap1 from EMSA. The percentage of DNA bound to TRF2 in experiment shown in part B was calculated as the relative change of intensity of the lower band normalized by the intensity signal of free DNA in protein absence (first lane on the left). (**E**) The release of telomeric DNA pre-bound with TRF2 after Rap1 addition measured by fluorescence anisotropy. Fluorescence anisotropy of Alexa Fluor 488 labeled DNA duplex R5 (7.5 nM) bound to TRF2 (open circle) after Rap1 addition (close circle) is shown. The vertical arrow depicts the moment when Rap1 was added instead of the initially added TRF2.

We used fluorescently labeled double-stranded human telomeric DNA duplexes, 17 bp (GTTAGGGTTAGGGTTAG; G-strand sequence) and 35 bp long (GTTAGGGTTAGGGTTAGGGTTAGGGTTAGGGTTAG) denoted as R2 (two telomeric repeats) and R5 (five telomeric repeats), respectively, along with 47 nt long DNA substrate containing 24 nt overhang (CCACACGTTAGGGTTA GGGTTAG***GGTTAGGGTTAGGGTTAGGGTTAG***, G-strand sequence; overhang is in bold italic), denoted as Ov (Figure 2A); for comparison purposes, the duplex denoted as N with the non-telomeric sequence (CATGACCAGCCATGATG) was used. One strand in the duplexes was synthesized with the 3′-end C6 aminoalkyl linker and labeled with Alexa Fluor 488 supplied by Life Technologies. The purification of duplexes was performed on an anion-exchange chromatography column Mono-Q HR 5/5 (GE Healthcare) in 0.1–2 M NaCl or LiCl gradient. The molar absorption coefficients of the single strands were determined based on sequence and contribution of fluorophores at 260 nm. The DNA oligonucleotides were supplied by Sigma-Aldrich or VBC Biotech (Vienna, Austria).

### Electrophoretic mobility shift assay

To describe TRF2 binding to telomeric DNA, electrophoresis in 5% non-denaturing polyacrylamide in 0.25×Tris-Borate-EDTA (TBE) buffer was used. Reactions containing the same amount of fluorescently labeled DNA (3 pmol) and increasing amounts of protein TRF2 (3–15 pmol) were prepared. DNA was labeled with fluorophore Alexa Fluor 488. The influence of protein Rap1 on DNA-binding affinity of TRF2 was detected in reaction mixtures composed of a constant amount of labeled DNA (5 pmol), a fixed amount of protein TRF2 (10 pmol) and increasing amounts of protein Rap1 (20–80 pmol). In both cases, the reactions were supplemented with buffer (50 mM NaCl, 50 mM sodium phosphate, pH 7.0) to a volume of 15 and 3 μl of 6× loading buffer (60% glycerol; 10 mM Tris/HCl, pH 7.6 and 60 mM ethylenediaminetetraacetic acid). Reaction mixtures were incubated for 15 min on ice and then loaded on horizontal 5% (w/v) non-denaturing polyacrylamide gels in 0.25×TBE buffer. The electrophoresis proceeded at 1 V/cm for 45 min and for an additional 3 h at 2 V/cm at 4°C. Fluorescently labeled DNA in gels were analyzed with a FLA 7000 imaging system (Fujifilm).

### Fluorescence anisotropy

FA measurements are based on excitation of fluorescently labeled molecule by linearly polarized light, subsequent change in molecular orientation and therefore different levels of polarization of fluorescence emission in perpendicular directions. FA is then represented as the ratio of the difference between the vertically and horizontally polarized light intensities and total emission intensity. Practically, FA value is relatively low for a small fast rotating fluorescently labeled DNA oligonucleotide. A protein bound to a fluorescently labeled DNA oligonucleotide slows down the rotation of the DNA molecule in solution. Thus, a FA value increases with the amount of protein–DNA or protein–protein complex formed. Value of FA is directly proportional to the fraction of protein–DNA complex. It has been confirmed experimentally using the fluorescently labeled DNA that a fluorescence intensity value has no significant effect on a FA value within interval of intensity measured during binding experiments (Supplementary Figure S6). A dissociation constant (*K*_d_) was determined from the curve representing the dependence of FA on the concentration of protein added to the solution in the cuvette. Fitting analyses were carried out using programs SigmaPlot 12 (Systat Software) and DynaFit4 (version 4.04.085; BioKin Ltd.) (24).

### Fluorescent labeling and spectroscopy of proteins

Rap1 was labeled with fluorescent dye Alexa Fluor 594 or 488. Subsequently, labeled proteins were separated from free fluorophores by gel filtration using a PD 10 Desalting Column (GE Healthcare). The experimental instrument setting for fluorescence measurement of Rap1 conjugated with the fluorophore Alexa Fluor 594 was 584 nm and 611 nm with the same width of slits 7 nm for excitation and emission. To measure TRF2 binding to DNA that was conjugated with the fluorophore Alexa Fluor 488, the excitation wavelength was set to 492 nm and emission wavelength to 516 nm with the width of slits 9 nm. The integration time was 3 s. The FA titration experiments were carried out in a 10×4 mm quartz-glass cuvette with chamber for magnetic bar stirrer. FA was measured at 25°C in the buffer containing 50 mM NaCl and 50 mM sodium phosphate (pH 7.0) if not stated otherwise. All fluorescence measurements were performed on a FluoroMax-4 spectrofluorometer (Horiba Jobin Yvon) with an L-format set up equipped with automatically adjustable polarizers for excitation and emission lights under control of an Origin-based FluorEssence software (version 2.1.6).

### Electrostatic component of binding

The contribution of electrostatic interactions was determined from the linear dependence of the binding constants on the increasing concentration of NaCl. The electrostatic component of binding originates from the formation of ion pairs between the cationic amino acid residues of the protein and the negatively charged phosphate groups of DNA or amino acid residues of the other protein. The electrostatic component of binding was determined from the binding constant dependence on ionic strength as described in (25) and in Supplementary Data.

### Isothermal titration calorimetry

ITC experiments were carried on a VP-ITC instrument (Microcal, GE Healthcare) at 25°C. Proteins were diluted in the same buffer 50 mM NaCl and 50 mM sodium phosphate (pH 7.0) and degassed. The cell (1423 μl) was filled with TRF2 (5 μM). Rap1 (44 μM) was added in 20 injections of 10 μl at 5 min intervals, with a stirring rate 240 rpm. Experimental data were analyzed in Origin 7.0 software using one-site binding model to fit a theoretical titration curve. Binding constant (*K*_a_), reaction stoichiometry (*n*) and binding enthalpy (Δ*H*) were obtained from the fit. Binding free energy (Δ*G*) and entropy change (Δ*S*) were determined from the equation:

(1) \begin{equation*} \Delta G = - {\rm R}T\ln \;K_{\rm a} = \Delta H - T\Delta S. \end{equation*}

### Surface plasmon resonance

Binding interactions between TRF2 and Rap1, TRF2 and DNA, complex Rap1–TRF2 and DNA and between Rap1 and DNA were analyzed using ProteOn (BioRad) on GLC and NLC sensor chips (BioRad) in a Phosphate Buffered Saline with Tween 20 (PBST). The detailed conditions of SPR measurements are available in Supplementary Data.

## RESULTS

### Full-length protein Rap1 binds to full-length TRF2 with high affinity and equimolar ratio

In order to analyze direct binding of full recombinant Rap1 to TRF2 in solution, ITC was employed. Three independent ITC titrations have been carried out. Rap1 was injected into an ITC cell containing TRF2 and heat exchange was measured (Figure 1B). Also control ITC injections have been carried out: injections of Rap1 in the cell containing buffer, the injections of buffer in the cell containing TRF2 and injections of buffer in the cell containing buffer (Supplementary Figure S2). The titration of Rap1 in the cell containing buffer has been used for a proper data normalization and baseline subtraction. The binding curve analysis showed rather high association constant 48 × 10^6^ per M and corresponding dissociation constant 21 nM (Figure 1B). A further analysis of the thermodynamic titration curve shows that the enthalpy contribution to the overall free energy of association prevails over the entropy change. It means that the binding is driven by enthalpy which relates to formations of new protein–protein bonds. The value of dissociation constant is relatively low showing a high affinity of Rap1–TRF2 association. The stoichiometry of Rap1–TRF2 interaction was obtained from the position of the inflection of ITC curve. The stoichiometry ratio Rap1:TRF2 is 1.1 ± 0.3 (Figure 1B), indicating an equimolar stoichiometry, i.e. the formation Rap1:TRF2 protein complex at ratio 1:1. Both proteins TRF2 and Rap1 form dimers as it has been suggested previously (16,18). Although the data do not allow us to determine a particular number of interacting protein subunits, we assume that TRF2 and Rap1 are preferentially in dimeric forms in solution. Collectively, the observed ITC data show high binding affinity of TRF2 and Rap1. Moreover, ITC results also indicate that the number of complex forming molecules of Rap1 and TRF2 is equal.

### Consistent dissociation constant values were obtained for the formation of Rap1–TRF2 complex from independent methods

The binding of full-length Rap1 to full-length TRF2 was analyzed quantitatively by two additional independent methods: FA (Figure 1C) and SPR (Supplementary Figure S4). In case of FA, TRF2 was allowed to bind to fluorescently labeled Rap1. A reversed titration was performed during SPR measurements when Rap1 was allowed to bind to immobilized TRF2. The dissociation constant for TRF2 binding to Rap1 obtained from FA was 43 nM. Despite the reverse experimental arrangement and slightly different reaction conditions, the observed dissociation constants obtained by SPR and FA are in accordance and consistent with the value obtained from the previous ITC measurement. We observed only moderate effect of salt concentration on Rap1 binding affinity in NaCl concentration range 50–140 mM (Supplementary Figure S3). Identical results were obtained also using proteins lacking the N-terminal His-tags and in the presence of dithiothreitol (DTT) (Supplementary Figure S5A and B). Thus, a binding affinity in the lower-nanomolar range was obtained using three independent quantitative methods.

### Rap1 induces partial release of TRF2 from telomeric double-stranded DNA

As Rap1 and TRF2 form a functional complex on telomeres *in vivo*, we asked how Rap1 affects the DNA-binding affinity of TRF2. Therefore, we incubated DNA duplex R5 (for sequence see Figure 2A) with constant amount of TRF2 and increasing amount of Rap1. In order to monitor the changes in DNA affinity after Rap1 binding, we used the saturating binding ratio TRF2 dimer:DNA known from the previous experiment in the absence of Rap1 (Figure 2C). At saturation conditions, the band corresponding to the free DNA duplex disappeared when the ratio TRF2 dimer:DNA was 3:1. In experiments with Rap1 present, the lower than saturation ratio (2:1) was used in order to describe how the DNA affinity of TRF2 is changed in the presence of Rap1 (Figure 2B). With increasing concentration of Rap1, we observed an increasing amount of free DNA. Electrophoretic mobility shift assay (EMSA) analyses revealed that the addition of protein Rap1 induced the release of TRF2 from telomeric DNA (Figure 2D). In order to confirm, that Rap1 does not bind DNA directly even in the highest concentration used in Figure 2B, we allowed Rap1 to bind telomeric DNA R2 and R5 (Supplementary Figure S7E and F). The EMSA analysis showed no direct DNA binding of Rap1 until stoichiometric ratio 40:1 (Rap1:DNA). Strikingly, in the presence of Rap1 the binding sites on DNA are not saturated by TRF2 compared to full saturation of binding sites if there is only TRF2 present. The EMSA results suggest that Rap1 decreases TRF2 binding affinity to telomeric double-stranded DNA.

Our previous results from gel electrophoresis assay suggest that Rap1 may disrupt preformed TRF2–DNA complex. In order to confirm the observations from EMSA experiments, we performed FA measurements. First, TRF2 was added to the solution containing fluorescently labeled DNA. The formation of TRF2–DNA complex was demonstrated by an increase of anisotropy value. Next, Rap1 was added to the solution, which led to an immediate drop in the anisotropy value (Figure 2E). The observed anisotropy decrease reflects the release of TRF2 from preformed TRF2–DNA complexes. Thus, direct anisotropy measurements confirmed that Rap1 induces TRF2 release from DNA duplex.

### Rap1 decreases the duplex DNA-binding affinity of TRF2

**Figure 3.**
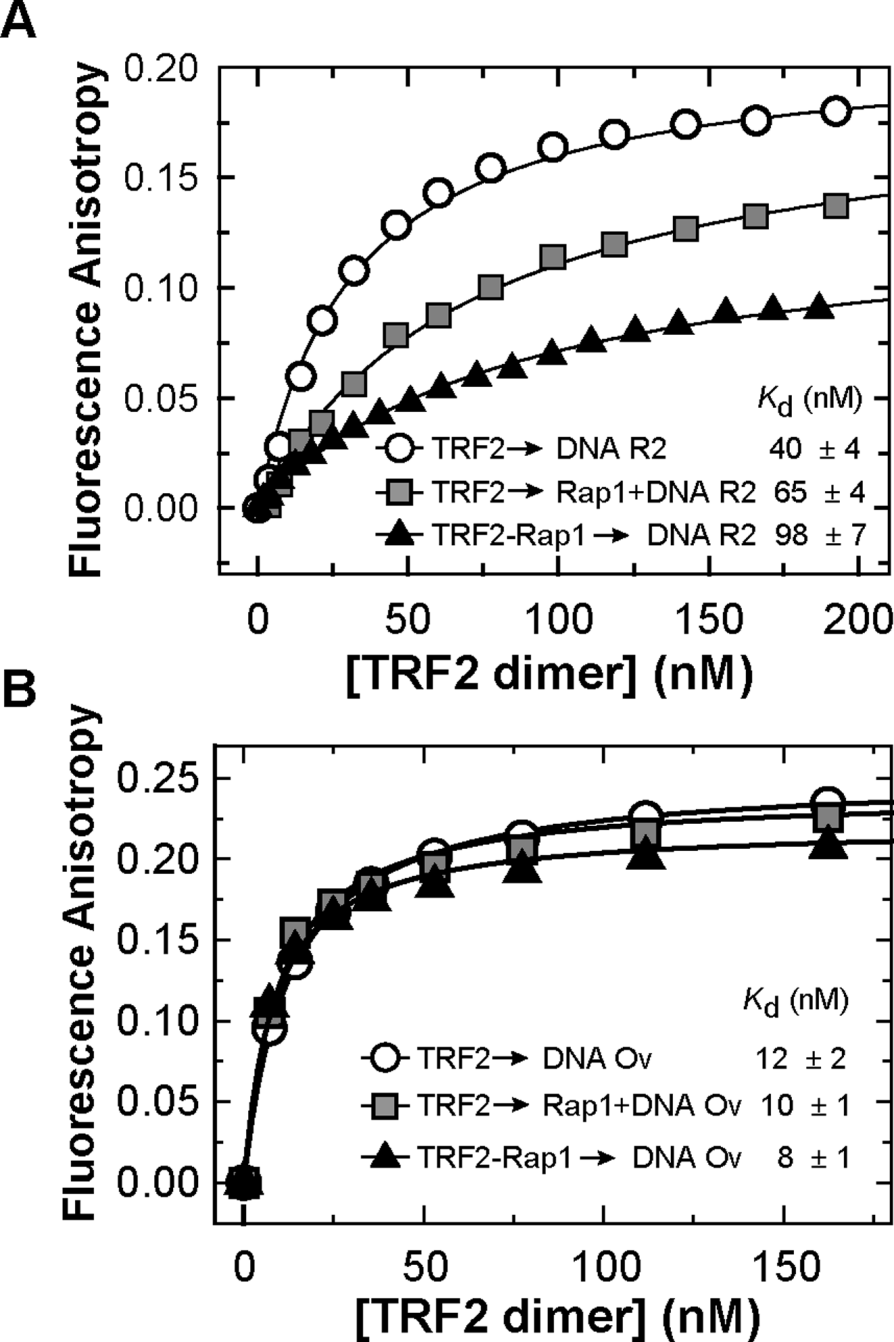
Rap1 decreases the binding affinity of TRF2 to telomeric DNA duplex but does not affect TRF2 binding to duplex/overhang junction. (**A**) Representative binding isotherms of TRF2 binding Alexa Fluor 488 labeled telomeric DNA duplex R2 (7.5 nM) in the presence of Rap1; measured by FA. Three plots show changes of TRF2 binding affinity to DNA in the absence (circle) or in the presence of Rap1 either in DNA mixture (square) or in a complex with TRF2 (triangle). (**B**) Binding isotherms of TRF2 with Alexa Fluor 488 labeled telomeric Ov DNA (7.5 nM) with overhang in the presence of Rap1 measured by FA. The symbol denomination is same as in part A. The values of dissociation constants were determined by non-linear least square fits using the equation FA = FA_MAX_*·c*/(*K*_d_+*c*) for one-site binding model. The *K*_d_ values were calculated as averages of at least three independent measurements with standard errors displayed.

To quantify the effect of Rap1 on TRF2 binding affinity to double-stranded DNA, we carried out three FA measurements with different experimental arrangements. In the first arrangement, TRF2 alone was allowed to bind to a fluorescently labeled DNA duplex R2 (for sequence see Figure 2A). In the second arrangement, TRF2 was allowed to bind to equimolar mixture of Rap1 and DNA. In the last set of experiments, the equimolar Rap1–TRF2 complex was initially allowed to form and subsequently, the complex was allowed to bind to DNA (Figure 3). The obtained dissociation constants showed that Rap1 decreased DNA-binding affinity of TRF2. The effect of Rap1 was more pronounced when Rap1–TRF2 complex was formed prior to telomeric DNA binding. The quantification of DNA binding of TRF2 in complex with Rap1 suggests that Rap1 decreases the TRF2 binding affinity more than 2-fold. Similarly, we observed a decrease in binding affinity of Rap1-bound TRF2 to the R2 duplex as compared to TRF2 alone by SPR (Supplementary Figure S8). In order to describe the effect of Rap1 on the kinetics of TRF2 binding to DNA, we performed SPR studies. Unfortunately, initial kinetic data showed multiple binding sites on the 27-bp telomeric fragment originally used. Therefore a precise fitting analysis of initial SPR data was challenging. When the DNA substrate was redesigned and shortened (Supplementary Materials) the values for dissociation rate of TRF2 from DNA (*k*_off_) were similar in the presence or absence of Rap1 (Supplementary Table S4). Similarly, our SPR data did not provide us with convincing results showing significant effect of Rap1 on TRF2 off-rate constants for fully hybridized duplex DNA nor DNA with 3′ overhang (data not shown). It might be caused by the short length of telomeric DNA that was required for our SPR experiments.

Direct Rap1 binding to DNA was not detected by SPR in the same experimental conditions as were used for TRF2 (Supplementary Figure S8). In accordance with our EMSA data (Figure 2B, the last lane on the right), in order to confirm that Rap1 affects specifically the DNA-binding of TRF2, we described the effect of Rap1 on DNA binding of TRF1, another DNA-binding shelterin protein. As Rap1 does not bind TRF1, there should be no significant effect of Rap1 on the DNA affinity of TRF1. Indeed, we observed no significant change in the dissociation constant of TRF1 binding to telomeric DNA in the presence of Rap1 (Supplementary Figure S9A).

### Rap1 does not affect TRF2 binding to the junction of duplex and overhang region of telomeric DNA

It has been shown that TRF2 preferentially binds to single/double-strand DNA junctions (26). In order to asses Rap1 effects on the affinity of TRF2 to such junctions, we allowed TRF2 to bind to a telomeric DNA substrate containing a 3′ single-stranded DNA overhang (Ov, see Figure 2A for sequence). The length of duplex part was 23 bp and the length of the single-stranded overhang was 24 nt. Importantly, we observed that TRF2 binding affinity to single/double-strand junction containing DNA Ov is more than 3-fold higher than TRF2 affinity for double-stranded telomeric DNA R2. Rap1 complexation with TRF2 caused no significant change of TRF2 binding affinity to Ov DNA, as all *K*_d_ values are close to each other within confidence interval. It means that Rap1 causes only negligible changes of TRF2 binding affinity to the duplex–overhang junction of telomeric DNA (Figure 3B). Additionally, our EMSA experiments showed no significant release of free DNA after Rap1 was added to TRF2 pre-bound to single/double-strand junction containing DNA Ov (Supplementary Figure S14B). These results suggest that Rap1 has no effect when TRF2 binds to single/double-strand junction regions of telomeric DNA.

### Rap1 increases TRF2 binding selectivity to telomeric DNA almost 2-fold

As Rap1 modulated the binding affinity of TRF2 to double-stranded telomeric DNA, we next asked whether this was also the case for non-telomeric DNA duplexes. In order to address the effect of Rap1 on TRF2 binding to non-telomeric DNA, we analyzed TRF2 interaction with telomeric R2 and non-telomeric DNA duplex N of the same length (17 bp) in the absence or presence of Rap1 (Supplementary Figure S10). At first, only TRF2 was allowed to bind DNA. Subsequently, the complex of TRF2 with Rap1 was used in DNA-binding assays. It was revealed that TRF2 binding affinity to telomeric DNA is approximately 5-fold higher in comparison with TRF2 binding affinity to non-telomeric DNA duplex N. In other words, the selectivity, which is defined as the ratio of association constant for protein binding to specific telomeric R2 and non-specific random N DNA, was five (Supplementary Figure S10). On the other hand, in the presence of Rap1, the binding affinity of TRF2 is more than 8-fold higher to telomeric than to non-telomeric DNA duplexes (Supplementary Figure S10); the selectivity was eight. Based on these results, we conclude that TRF2 in complex with Rap1 binds telomeric DNA with nearly 2-fold higher selectivity.

### Rap1 improves the selectivity of TRF2 for telomeric DNA by reduction of non-specific electrostatic interactions

**Figure 4.**
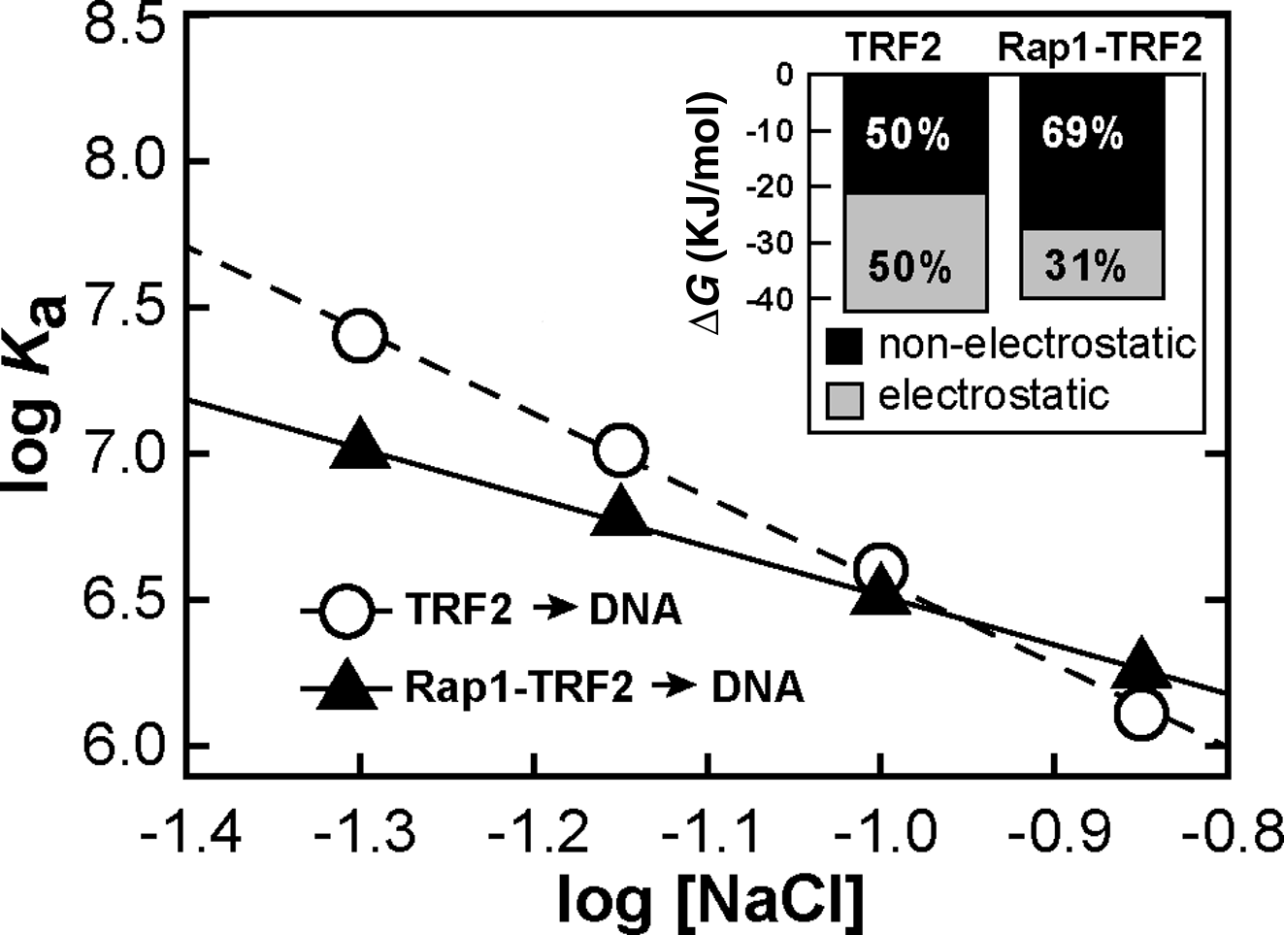
Rap1 reduces electrostatic attraction of TRF2 to telomeric DNA. Dependences of the association constants for binding of TRF2 and Rap1–TRF2 complex to telomeric DNA duplex R2 (7.5 nM) on NaCl concentration. The sodium phosphate buffer (50 mM; pH 7.0) contained NaCl in concentration range 50–140 mM. The inset with the bar graph shows the relative contribution of electrostatic and non-electrostatic interactions to free energy of binding of TRF2 or Rap1–TRF2 to telomeric DNA calculated from the linear salt dependence of the association constant logarithms.

In order to address the molecular origin of enhanced telomeric DNA recognition of TRF2 induced by Rap1, we carried out set of DNA affinity measurement of TRF2 in different salt conditions. To analyze the electrostatic component of TRF2 interactions with telomeric DNA in the presence or absence of Rap1, two independent sets of binding affinity measurements were performed. Dissociation constants of TRF2 binding to DNA were determined using buffers containing NaCl concentrations ranging from 50 to 140 mM. Average values of association constants (*K*_a_) were calculated as reciprocal values of dissociation constants obtained for at least three independent measurements at each salt condition. Logarithms of *K*_a_ were plotted against logarithms of salt concentration (Figure 4). The linear dependence of log *K*_a_ on the logarithm of the NaCl concentration indicates that electrostatic interactions are involved in the TRF2 binding to DNA. From the linear regression, the parameter *Z*, corresponding to number of newly formed ion pairs, and the value of log *K*_a_^nel^, corresponding to binding affinity arisen from specific (non-electrostatic) interactions, were identified (Supplementary Table S2). The parameter *Z* revealed that the binding of TRF2 to telomeric DNA resulted in the formation of approximately five ion pairs in average. On the other hand, in the presence of Rap1, TRF2 formed only three ion pairs with DNA. The decreased *Z* value means that the electrostatic interactions with DNA were less pronounced when the preformed complex of Rap1 and TRF2 bound DNA. The contribution of electrostatic component to overall DNA-binding affinity of TRF2 was calculated from values corresponding to the total binding affinity *K*_a_ and non-electrostatic contribution to the binding affinity *K*_a_^nel^. Based on the salt dependence of association constant, the total affinity and corresponding Gibbs free energy of binding was divided into contributions originated from electrostatic and non-electrostatic interactions (Inset of Figure 4, Supplementary Table S3). It can be concluded that the non-electrostatic interactions (specific in their origin) contribute by 50% to the total energy of binding of TRF2 and by approximately 69% for complex Rap1–TRF2. Therefore, Rap1 decreases the electrostatic component of binding by more than one third. Consequently, Rap1 induced 19% relative increase of contribution of the non-electrostatic interactions between TRF2 and DNA, which are mainly specific in their nature. Thus, Rap1 reduces non-specific electrostatic interactions and as a result improves TRF2 selectivity to telomeric DNA.

As TRF2 contains N-terminal basic domain, we speculated that positively charged residues of this domain mainly contribute to the non-specific dsDNA binding of TRF2. Therefore, negatively charged Rap1 might act by counteracting this positive charge of TRF2. If our reasoning is correct, Rap1 should add a higher net negative charge to TRF2 lacking the basic N-terminal residues (TRF2ΔB) than to the full-length protein and therefore induce a more profound displacement of TRF2ΔB from duplex DNA than in case of full-length TRF2. To test this idea, we allowed either full-length TRF2 or TRF2ΔB to bind to DNA R2 until about half saturation range was achieved (Supplementary Figure S11). When Rap1 was added, we observed that significantly more DNA was released from TRF2ΔB than in case of full-length TRF2. This experiment strongly supports our previous measurements and conclusions about the effect of Rap1 on DNA-binding affinity of TRF2 and the importance of the TRF2 basic domain to non-specific DNA binding.

## DISCUSSION

We revealed the molecular mechanism of how Rap1 affects TRF2 binding to DNA by using a combination of quantitative biophysical approaches. The explanation of the origin of selective DNA binding of shelterin protein TRF2 is essential for understanding shelterin functions in genome stability maintenance in humans and mammals. Moreover, our findings could be applied on proteins that bind DNA and participate in gene regulation through selective DNA recognition. The quantitative descriptions include several new observations that provide a more complete understanding of the activity of the critical shelterin subunit TRF2 on DNA, which extents and confirms the previous separate observations of other investigators (11,17,18).

In this study we have quantified direct interactions of full-length Rap1 and TRF2 (Figure 1). Additionally, we assessed how Rap1 affects the affinity and selectivity of protein TRF2 for telomeric DNA. The observed absolute *K*_d_ value for binding of full-length Rap1 and TRF2 is in a very good correlation with the value of *K*_d_ for the binding of C-terminal domain of human Rap1 (1,17). Our ITC and EMSA data revealed Rap1:TRF2 ratio 1:1, i.e. the same ratio as has been shown for truncated variants of Rap1 by ITC and by gel chromatography previously (17,18). The observed binding ratio supports the explanation that one Rap1 dimer binds one TRF2 dimer in solution.

In order to address the possible direct interaction of Rap1 with telomeric DNA duplexes, we allowed Rap1 bind to telomeric DNA duplexes. Our SPR and FA data showed no significant binding of Rap1 to telomeric DNA (Supplementary Figures S8 and S12, respectively). Additionally, our EMSA experiments have shown that Rap1 does not bind telomeric DNA until Rap1:DNA molar ratio 40:1 (Supplementary Figure S7E and F). These results are partially contradicting the previous finding that Rap1 binds telomeric DNA directly (18). The difference might be caused by shorter length of DNA duplexes and different binding conditions used in both types of experimental studies. Some differences might be also attributed to different protein expression systems used in both studies (bacterial expression in case of this study versus baculovirus expression in insect cells).

The main objective of our study was to quantify how Rap1 affects affinity and selectivity of protein TRF2 to telomeric DNA. We found that, on one hand, the presence of Rap1 increases the selectivity of TRF2 binding to telomeric DNA by 2-fold, but on the other hand, Rap1 decreases TRF2 binding affinity to DNA by approximately 2-fold.

The body of evidence that Rap1 improves TRF2 selectivity to telomeric DNA is that the dissociation constants ratio for TRF2 binding to telomeric and non-telomeric DNA was increased 2-fold in the presence of Rap1 (Supplementary Figure S10). The observed selectivity increase is in a very good accordance with the results obtained recently from gel retardation assays by Arat and Griffith (18). Additionally, when we studied Rap1 effect on DNA binding of TRF2, we found that Rap1 induced TRF2 release from telomeric DNA duplexes. The first direct proof that Rap1 disrupts preformed TRF2–DNA complexes was that the intensity of the electrophoretic band corresponding to free DNA was increased after the addition of Rap1 into mixture of TRF2 pre-incubated with DNA (Figure 2B and C). Importantly, we also observed a FA drop when Rap1 was titrated into TRF2 pre-bound to fluorescently labeled dsDNA (Figure 2E). Of note, the difference in the percentage of TRF2–DNA complex loss determined by EMSA and FA (Figure 2D and E) might be caused by 8-fold higher DNA concentration in case of EMSA experiments compared to FA measurements. TRF2–DNA complex is thermodynamically more stable at concentrations above *K*_d_ which could cause the less pronounced Rap1 effects on total release of DNA in case of EMSA experiments (Figure 2B). The more intensive Rap1 effect on DNA release was observed when the concentration of DNA in EMSA experiments was decreased (Supplementary Figure S13). Together, the observed FA decrease and EMSA detection of increased ratio of free DNA after Rap1 addition strongly support the view that Rap1 causes the partial release of TRF2 from the DNA duplex.

A probable explanation of how Rap1 could contribute to the observed reduction in the DNA-binding affinity of TRF2 is a modification of net surface charge after Rap1–TRF2 complex formation. Protein interaction with DNA occurs in two steps. In the first step an electrostatic, non-specific attraction of interacting partners occurs (27). In the second step a non-electrostatic, sequence-specific binding based on newly formed interactions takes place (28,29). In order to address the net charge influence of Rap1 we determined the dependence of DNA-binding affinity of TRF2 on ionic strength with and without Rap1 pre-incubated with TRF2. The less pronounced salt dependence of DNA-binding affinity for complex Rap1–TRF2 revealed the net surface charge was neutralized significantly after the assembly of Rap1 and TRF2. Based on our quantitative measurements, we propose that the charge neutralization together with possible allosteric changes of DNA interacting surface of TRF2 after Rap1 binding are main origins of the improved selectivity of TRF2 binding to telomeric DNA.

**Figure 5.**
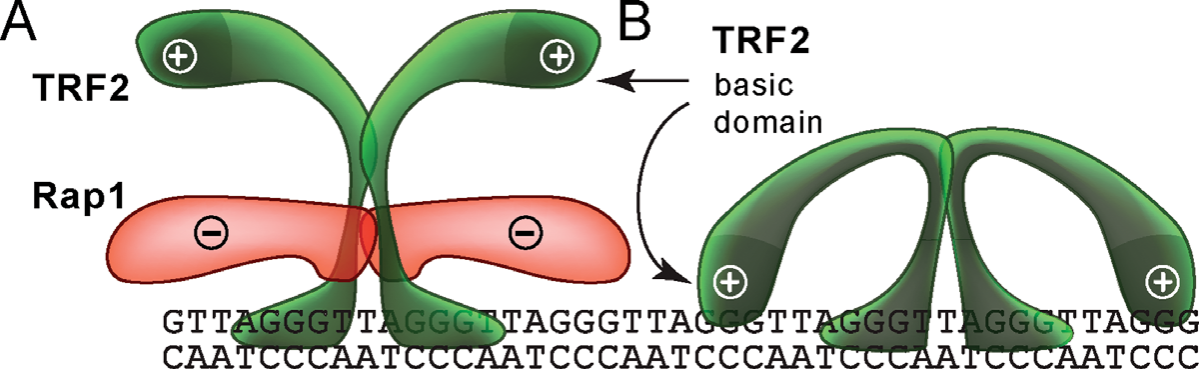
A possible mechanism of how Rap1 affects the TRF2 binding to telomeric DNA. (**A**) Negatively charged Rap1 induces the neutralization of positively charged TRF2 and prevents interaction of TRF2 basic domain with DNA. (**B**) Additional TRF2 binding to DNA via the basic N-terminal domain may occur and is sterically allowed when Rap1 is absent.

Eventually, we assessed how Rap1 affects TRF2 binding to DNA substrate Ov containing naturally occurring overhang that comprises four telomeric repeats (Figure 2A). We observed that Rap1 positively stimulated TRF2 binding to single/double-strand junction of telomeric DNA (Figure 3B). The different effect of Rap1 on TRF2 binding to full DNA duplex and overhang containing DNA substrates could be closely connected with DNA-binding contribution of positively charged basic domain of TRF2. We speculate that Rap1, after binding to TRF2, might shield the positively charged basic domain on the N-terminus of TRF2 from mainly non-specific electrostatic interaction with DNA. This view is supported by theoretical overall negative charge of Rap1 (pI 4.6) and positive charge of TRF2 (pI 9.2) in a buffer with pH 7. A scheme of the putative shielding effect of Rap1 is shown in Figure 5.

Moreover, the interaction model takes into consideration dimeric arrangements of TRF2 and Rap1. The sequence selective binding of TRF2 in dimeric form is in agreement with DNA sequence recognition mechanism of other selectively binding proteins that take part in regulatory mechanisms (30).

To test this model we prepared a truncated variant of TRF2 lacking the N-terminal basic domain. In accordance with our hypothesis, when the basic domain is absent, TRF2 should have a significantly lowered DNA affinity. Indeed, when we measured the DNA-binding affinity of TRF2 lacking the basic domain, DNA-binding affinity was decreased to the level of the affinity observed for the binding of TRF2 with Rap1 present (Supplementary Figure S9B). Moreover, we observed that more telomeric double-stranded DNA was released when Rap1 added to TRF2 lacking the basic domain pre-bound to DNA compared to full-length TRF2 (Supplementary Figure S11). This finding strongly supports our previous measurements and the proposed model of Rap1 effects on DNA-binding affinity of TRF2.

It has been previously demonstrated that the basic domain of TRF2 participates in DNA binding and stabilizing of opened intermediates of telomeric DNA (31), as Poulet *et al*. have shown by combination of nuclear magnetic resonance, differential scanning calorimetry (DSC) and permanganate probing experiments. It has been suggested by the same laboratory that TRF2 basic domain has evolved to finely regulate TRF2 ability to condense DNA (32). Additionally, it has been suggested that the basic domain of TRF2 facilitate and stabilize special arrangements of DNA strands into functional t-loops (9) and TRF2 stabilization of such DNA arrangements is compromised when the basic domain of TRF2 is absent.

Thus, our data together with findings of other laboratories about TRF2 basic domain contribution to DNA binding suggest that the decreased DNA-binding affinity of TRF2 lacking the N-terminal basic domain supports the view that TRF2 binding to DNA via the basic domain is diminished in Rap1 presence.

The detailed analysis of contributions of the basic domain to DNA binding of TRF2 is subject of our future studies in order to quantify its effect on non-specific electrostatic interaction with DNA. Importantly, the functional and possibly also structural changes of TRF2 upon binding of Rap1 might be directly connected with a higher capacity of complex Rap1–TRF2 to fold telomeric DNA into loops as has been shown by Arat and Griffith (18). Moreover, it has been observed that Rap1 is able to disturb higher oligomeric arrangement of TRF2 (16,22,28). The ability of Rap1 to modulate DNA binding of TRF2 could be crucial in processes during which Rap1 affects localization of TRF2 on DNA strand and this way helps to acquire an optimal distribution of TRF2 and whole shelterin protein complexes on DNA.

In order to put our findings using TRF2 and Rap1 expressed bacterially into the context with other studies using insect cell expression, we assessed how different expression systems affect multimer distribution of the proteins. We have compared bacterially produced proteins and proteins expressed in insect cells using electrophoresis at mild denaturing conditions (data not shown) (33). The only observed difference was that TRF2 produced in insect cells showed a slightly greater ratio of multimeric forms than TRF2 produced in bacterial cells. The greater tendency of TRF2 to form multimers might be connected with the different translational modifications in bacterial and insect expression systems. The multimeric patterns of Rap1 expressed in bacterial and insect cells were almost identical.

Rap1 contribution to the whole shelterin function and signal pathways of the cell is matter of a long discussion (10,13,16). The evolutional conservation of Rap1 as part of human and mice shelterin complex is intriguingly pointing toward the possible importance of Rap1 presence within shelterin (11,13). Based on the findings obtained by our *in vitro* binding studies, one may speculate that Rap1 is needed for efficient directing of TRF2 to its proper binding location at telomeres also *in vivo*. TRF2 alone could be accumulated in internal chromosomal or peritelomeric regions. After Rap1 binding, Rap1–TRF2 complex might relocate to a single/double-strand junction of telomeric DNA. Although little to no effect on TRF2 telomere binding is observed after Rap1 deletion in both human and mouse cells (13,19), it is possible that other shelterin components (such as TRF1-bound TIN2) might contribute to proper TRF2 binding, perhaps in a semi-redundant manner with Rap1. This alteration in relocalization effect on TRF2 could explain why Rap1 is dispensable part of shelterin complex. Indeed, a partial destabilization of telomere-bound TRF2 is observed in mouse cells lacking TIN2 (34). It would be therefore interesting to study the effect of Rap1 on TRF2 binding to telomeres in TIN2-deficient cells and in the context of other shelterin subunits.

We conclude that Rap1 serves as a selectivity enhancer of TRF2 with newly found ability to partially remove bound TRF2 from telomeric DNA duplex. The observed Rap1 release activity suggests that Rap1 plays an important role in tuning DNA interactions of TRF2, the central subunit of shelterin protein complex. Our data showing the release of TRF2 from telomeric DNA in the presence of Rap1 suggest that protein Rap1 might prompt the relocation of TRF2 to the preferred single/double-strand junction of telomeric DNA. For the first time here, we used combination of quantitative biophysical approaches to describe and explain molecular origins of Rap1 contribution to selective TRF2 recognition of telomeric DNA. We found that Rap1 neutralizes the electrostatic attraction of TRF2 to DNA. Hence, Rap1 reduces overall DNA-binding affinity of TRF2 in order to improve its binding selectivity toward telomeric DNA. The following studies focused on shelterin protein dynamics using single molecule approaches will be of particular interest as they can reveal relocation dynamics of TRF2 induced by Rap1 and the mechanism of regulation of shelterin binding and distribution on telomeres.

## SUPPLEMENTARY DATA

Supplementary Data are available at NAR Online.

SUPPLEMENTARY DATA
