# Supplementary material for: Human Rap1 modulates TRF2 attraction to telomeric DNA
Source: Nucleic Acids Res. 2015 Feb 11;43(5):2691–700. doi: 10.1093/nar/gkv097 (PMC4357705; doi:10.1093/nar/gkv097)
Supplement: SUPPLEMENTARY DATA [file supp_43_5_2691__index.html]

Human Rap1 modulates TRF2 attraction to telomeric DNA — SUPPLEMENTARY DATA 

# Human Rap1 modulates TRF2 attraction to telomeric DNA

## SUPPLEMENTARY DATA

**Files in this Data Supplement:**

- Supplementary Materials and Methods
