# Supplementary material for: Human Rap1 modulates TRF2 attraction to telomeric DNA
Source: Nucleic Acids Res. 2015 Feb 11;43(5):2691–700. doi: 10.1093/nar/gkv097 (PMC4357705; doi:10.1093/nar/gkv097)
Supplement: SUPPLEMENTARY DATA [file supp_gkv097_nar-00072-x-2015-File007.pdf]

Supplementary Materials and Methods

Human Rap1 Modulates TRF2 Attraction to Telomeric DNA

Eliška Janoušková, Ivona Nečasová, Jana Pavloušková, Michal Zimmermann, Milan Hluchý, Victoria Marini, Monika Nováková, and Ctirad Hofr

Electrostatic contribution to binding affinity

The number of ion pairs formed upon protein–DNA binding and corresponding electrostatic contribution to overall binding affinity (K_a) could be derived from the dependence of the binding constant on salt concentration according to the equation:

$$\log K_a = \log K_a^{nel} - Z \bullet \varphi \bullet \log[\mathrm{NaCl}] \qquad (1)$$

where K_a is association constant representing the overall affinity, K_a^{nel} is non-electrostatic component of association constant, Z is the number of DNA phosphates that interact with the protein, φ is the number of Na^+ cations released from the interaction with the phosphate group after binding of protein to DNA. For double-stranded B-DNA molecule with a length of 24 bp and shorter, the value for φ is approximately 0.64 (26). The association constant (K_a) were calculated as the reciprocal value of the dissociation constant. From the association constant is possible to calculate the value of the Gibbs free energy according to the equation:

$$\Delta G_a = -2.3\ \mathrm{R}T \bullet \log K_a \qquad (2)$$

The binding energy could be divided into electrostatic and non-electrostatic terms:

$$\Delta G_a = \Delta G_a^{nel} + \Delta G_a^{el} \qquad (3)$$

The electrostatic component disappears when the salt concentration approaches 1 M and the overall energy of binding is given only by the non-electrostatic component:

$$\Delta G_a^{nel} = -2.3\ \mathrm{R}T \bullet \log K_a^{nel} \qquad (4)$$

Surface plasmon resonance analysis

Protein-protein interaction between TRF2 and Rap1 was analyzed on the ProteOn GLC sensor chip (Bio-Rad). Protein TRF2 in concentrations from 10 to 2.5 µg/ml was immobilized on the chip in a PBST buffer onto five of the six ligand channels using the amine coupling reagents 1-ethyl-3-(3-dimethylaminopropyl)-carbodiimide hydrochloride (EDAC, 133 mM) and N-hydroxysulfosuccinimide (sulfo-NHS, 33 mM). The sixth channel was not modified and served as a reference channel. Remaining unmodified surface groups were deactivated with 1 M ethanolamine HCl, pH 8.5. A sample of running buffer was used for ligand injection in the reference channel. For analyte binding, five solutions of Rap1 were prepared at concentrations of 80, 40, 20, 10 and 5 nM by serial dilution in PBST buffer. The sixth channel was used as reference channel using only the running buffer. The samples were

injected for 240 s at a flow rate of 100 µl/min; dissociation time was 1200 sec. The obtained sensorgrams were processed for baseline alignment and reference channels subtraction (Figure S4 A). Equilibrium response levels were plotted against the analyte concentration. The equilibrium response plot was fitted using a simple bimolecular equilibrium model (Figure S4 B), from which the equilibrium dissociation K_d was determined.

DNA oligonucleotides used in SPR studies

Three different double-stranded oligonucleotides were used for SPR analyses. DNA duplex SR2 containing two human telomeric repeats 5'-TTAGGG-3' was prepared by annealing the 5' end biotinylated oligonucleotide (oligo) 5'-CTAACCCTAACCCTAAGTTAG-3' to the oligo 5'-CTAACTTAGGGTTAGGGTTAG-3'. DNA duplex Overhang_SR2 which consisted of a 21-bp stem with a 6 nucleotide overhang was prepared by annealing the 5' end biotinylated oligo 5'-CTAACCCTAACCCTAAGTTAG-3' to the oligo 5'-CTAACTTAGGGTTAGGGTTAGGGTTAG-3'. DNA duplex SN2 with random nontelomeric sequence was prepared by annealing the 5' end biotinylated oligo 5'-GATGTTCACAGAGTCAGTTAG-3' to the oligo 5'-CTAACTGACTCTGTGAACATC-3'. All complementary oligonucleotides were annealed in 20 mM Tris, 50 mM NaCl, pH 7.0 by heating for 5 min at 95 °C and cooled down to 25 °C by 1 °C per min for 70 minutes. Annealed double-stranded oligonucleotides were diluted in buffer and purified on an anion-exchange column using high-performance liquid chromatography. All oligonucleotides used in SPR studies were supplied by VBC Biotech (Vienna, Austria).

Protein-DNA interactions between TRF2 and DNA, complex Rap1-TRF2 and DNA, and Rap1 and DNA were analyzed at the flow rate of 70 µl/min. Biotinylated double-stranded oligonucleotides SR2, overhang_SR2 and SN2 were immobilized on the ProteOn NLC sensor chip in PBST buffer onto five of the six ligand channel surfaces using a 3 nM DNA concentration. The sixth channel served as a reference channel. A sample of running buffer was used for ligand injection in the reference channel. For analyte binding, five solutions of proteins TRF2, complex Rap1-TRF2 and Rap1 were prepared at concentrations of 80, 40, 20, 10 and 5 nM by serial dilution in PBST buffer. Complex Rap1-TRF2 was prepared in 1:1 molar ratio. The sixth channel was used as a reference channel using only the running buffer. The samples were injected for 350 s at a flow rate of 70 µl/min; the dissociation time was 900 s. The obtained sensorgrams were processed for baseline alignment and reference channels subtraction (Figure S78). Kinetic analyses were performed for the five analyte concentrations. Values of rate dissociation constant k_d (k-off) was obtained from concentrations of analyte, which gave the best response signal (Table S4).

Table S1. Primers used to prepare plasmid constructs for expression of Rap1 and TRF2.

Primer	Cloned sequence	Sequence of primer 5' – 3'
1 forward1	**Rap1**	CTGGAAGTTCTGTTCCAGGGGCCCATGGCGGAGGCGATGGATTTGGGC
2 forward 2	**Rap1**	GGGGACAAGTTTGTACAAAAAAGCAGGCTCCATGGCGGAGGCGATGGATTTGGGC
3 reverse	**Rap1**	GGGGACCACTTTGTACAAGAAAGCTGGGTTCATCATTTCTTTCGAAATTCAATCCTCCG
4 forward 1	**TRF2**	CTGGAAGTTCTGTTCCAGGGGCCCATGGCGGGAGGAGGCGGGAGTAGC
5 forward 2	**TRF2**	GGGGACAAGTTTGTACAAAAAAGCAGGCTCCCTGGAAGTTCTGTTCCAGGGGCCC
6 reverse	**TRF2**	GGGGACCACTTTGTACAAGAAAGCTGGGTTCATCAGTTCATG CCAAGTCTTTTCAGTGT

Table S2. Summary of measured and calculated binding constants for interaction of TRF2 and Rap1-TRF2 complex with DNA duplex R2 at different concentrations of NaCl.

TRF2 -> DNA

[NaCl] mM	Log [NaCl]	Aver. Value K_d (nM)	K_a ($10^6 M^{-1}$)	Log K_a	Δlog K_a/ Δlog [NaCl]	Log K_a^{nel}	Z
**50**	-1.30	40	25.0	7.4	-2.8	3.7	4.5
**70**	-1.15	98	10.2	7.0			
**100**	-1.00	250	4.00	6.6			
**140**	-0.85	780	1.30	6.1			

Rap1-TRF2 -> DNA

[NaCl] mM	Log [NaCl]	Aver. Value K_d (nM)	K_a ($10^6 M^{-1}$)	Log K_a	Δlog K_a/ Δlog [NaCl]	Log K_a^{nel}	Z
**50**	-1.30	98	10.2	7.0	-1.7	4.8	2.6
**70**	-1.15	170	6.0	6.8			
**100**	-1.00	320	3.2	6.5			
**140**	-0.85	550	1.8	6.3			

All presented equilibrium constant values are presented as means of at least three independent measurements. The relative standard error was less than 5%.

Table S3. Contribution of electrostatic ΔG^{el} and non-electrostatic ΔG^{nel} component to the total free energy for TRF2 binding to DNA duplex R2.

TRF2 -> DNA		Rap1-TRF2 -> DNA	
ΔG [kJ·mol^{-1}]	-42.2	ΔG [kJ·mol^{-1}]	-40.0
ΔG^{nel} [kJ·mol^{-1}]	-21.2	ΔG^{nel} [kJ·mol^{-1}]	-27.6
ΔG^{el} [kJ·mol^{-1}]	-21.0	ΔG^{el} [kJ·mol^{-1}]	-12.4

The relative standard errors of all presented mean values were less than 5%.

Table S4. Kinetic analyses of dissociation rate constants of TRF2 from DNA duplex SR2 with and without Rap1 present.

**TRF2 -> DNA**				
**protein (nM)**	**DNA (nM)**	**k_{off} (sec^{-1})**	**R_{max} (RU)**	**Chi^2**
80	2	8.0E-05	960	3.8
40	2	1.6E-04	670	6.7
20	2	3.4E-04	340	8.9
10	2	4.0E-04	200	6.0
5	2	4.6E-04	130	4.2
**Rap1-TRF2 -> DNA**				
**protein (nM)**	**DNA (nM)**	**k_{off} (sec^{-1})**	**R_{max} (RU)**	**Chi^2**
80	2	8.7E-05	1020	10.0
40	2	1.5E-04	700	11.0
20	2	2.1E-04	460	9.6
10	2	2.7E-04	300	6.7
5	2	3.1E-04	180	4.0

Values of k_{off} were obtained by fitting of dissociation part of sensorgrams in Figure S8 from the starting value Rmax of response unit (RU). Ch^2 is Chi-Square goodness of fit.

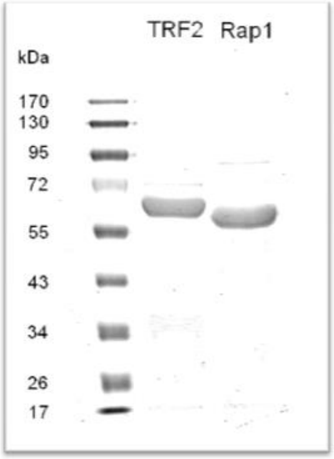

Figure S1. Verification of purity of TRF2 and Rap1 by SDS-PAGE. Separation was performed using a 10% SDS-polyacrylamide gel. From left: M, protein marker PageRuler™ Prestained Protein Ladder #SM0671 (Fermentas) (5 µl); TRF2 after IMAC (15 µg); Rap1 after IMAC (15 µg).

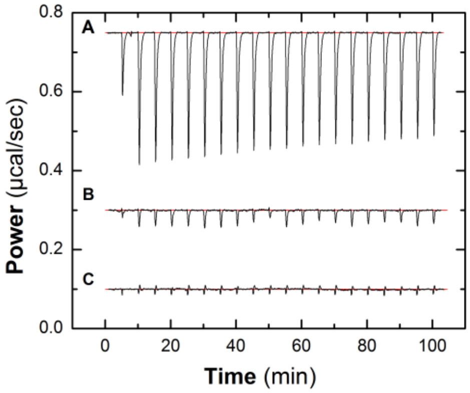

Figure S2. Control ITC titrations for normalization of TRF2 and Rap1 interaction. Injector solution was titrated in 20 injections of 10 µl into the cell with a stirring rate of 240 rpm: A) Injections of Rap1 (44 µM) into the cell containing buffer, B) injections of buffer into the cell containing TRF2 (5 µM) and C) injections of buffer into the cell containing buffer. The buffer used for ITC measurements was 50 mM NaCl and 50 mM sodium phosphate (pH 7.0). All measurements were performed at 25°C.

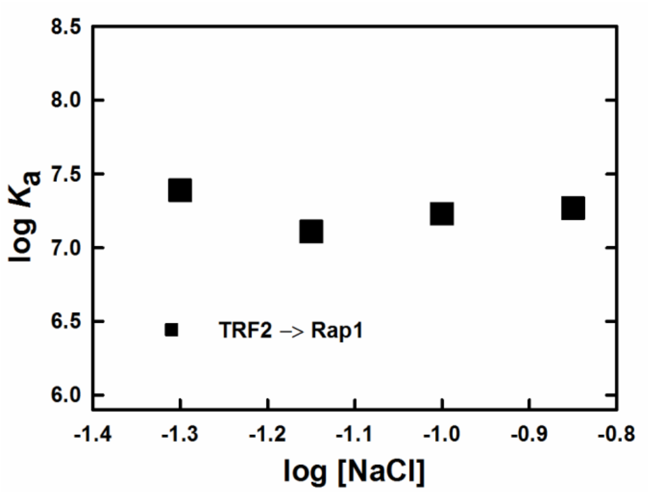

Figure S3. Salt concentration dependence of the association constant for binding TRF2 to Rap1. TRF2 was allowed to bind with Rap1 labelled by Alexa Fluor 594. The concentration of Rap1 was 100 nM. The sodium phosphate buffer (50 mM; pH 7.0) contained NaCl in concentration range 50-140 mM. Measurement conditions were the same as described in materials and methods above. Note that the binding affinity has not changed significantly in the salt concentration range.

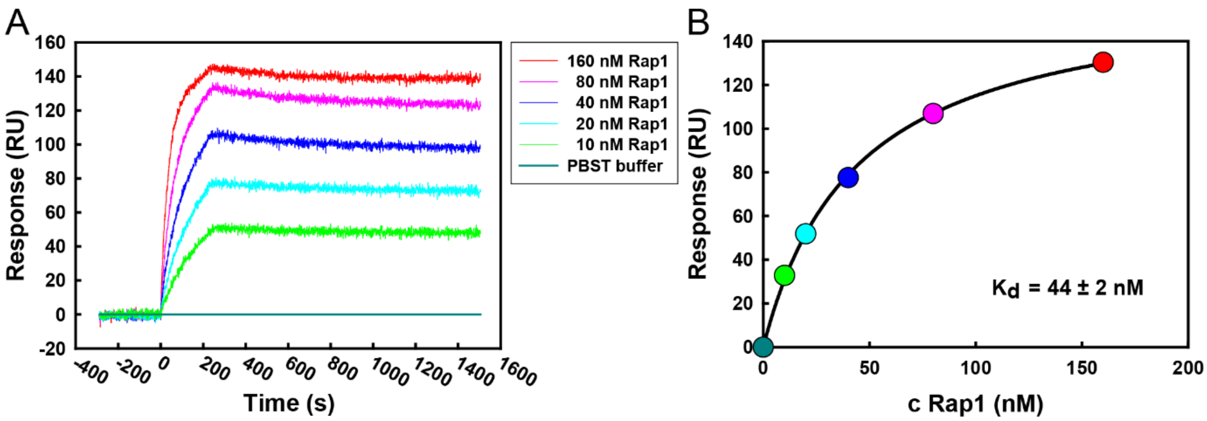

Figure S4. Binding of protein Rap1 to immobilized protein TRF2. Response maxima from the sensorgram (A) have been used to determine equilibrium dissociation constant K_d (B). RU, response units. See Supplementary Material and Methods for details.

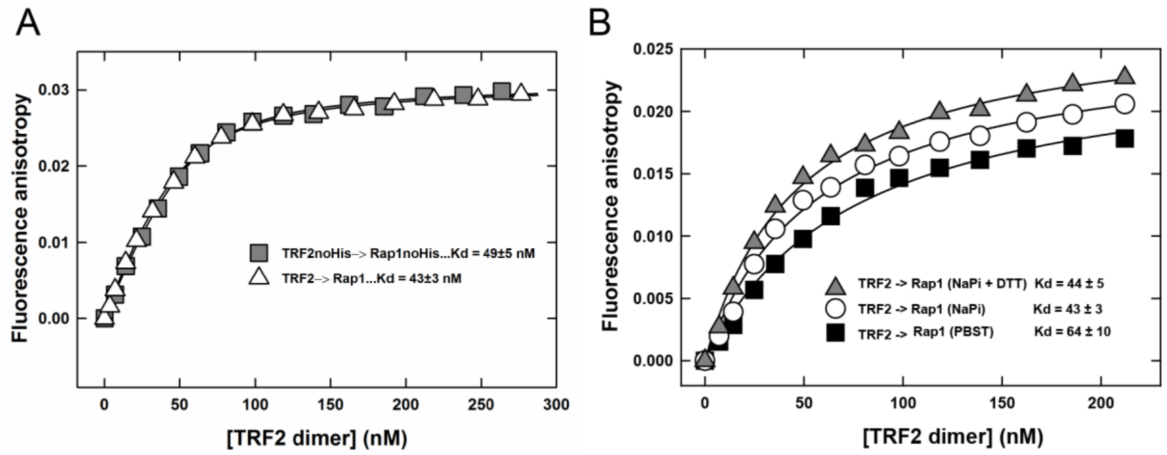

Figure S5. TRF2 binding affinity to Rap1 is not affected by His tags (A) nor DTT (B). Protein Rap1 was labelled with Alexa Fluor 594 and Rap1 without His-tag (Rap1noHis) with Alexa Fluor 488. The concentration of Rap1 and Rap1noHis was 100 nM. NaPi denotes solution of 50 mM NaCl in 50 mM sodium phosphate buffer (pH 7.0), DTT denotes 1 mM Dithiothreitol and PBST denotes Phosphate Buffered Saline with Tween 20 (~140 mM NaCl). The values of dissociation constants were determined by non-linear least square fits.

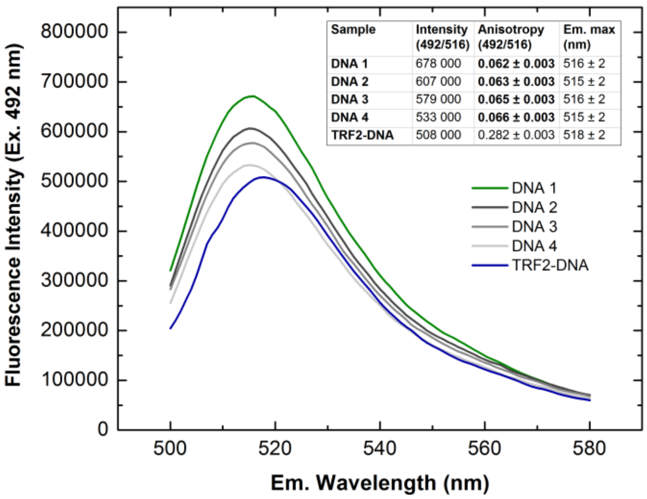

Figure S6. Fluorescence anisotropy values are independent on fluorescence intensity values in the whole measuring range. Emission fluorescence spectra of DNA R2 labelled by Alexa Fluor 488 were recorded at 8.7 nM (DNA 1), 8.6 nM (DNA 2), 8.5 nM (DNA 3), 7.8 nM (DNA 4) and 7.0 nM in presence of TRF2 (TRF2- DNA) at concentration 340 nM (full binding saturation). Excitation wavelength was 492 nm, excitation and emission slits were 4 nm. The inset table contains values of fluorescence intensity at 516 nm, values of fluorescence anisotropy measured at emission wavelength 516 nm using same excitation and emission slits 7 nm, and emission maxima.

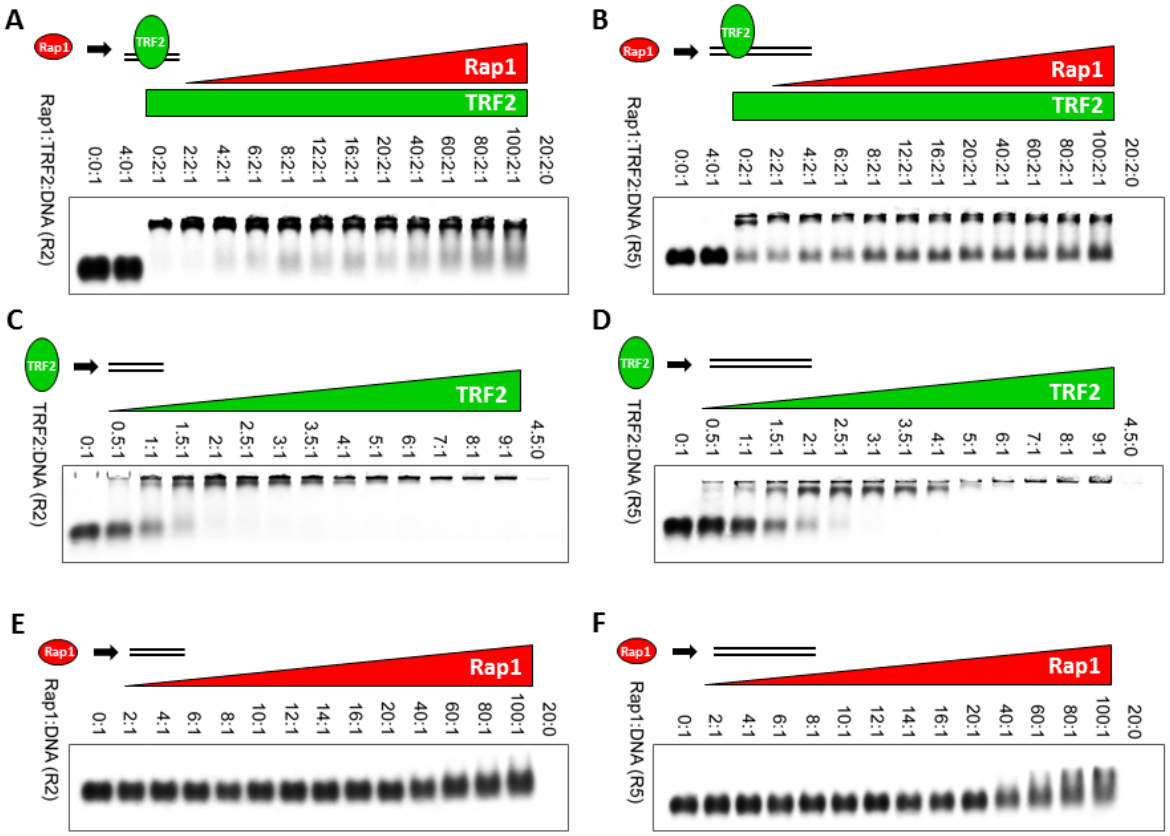

Figure S7. TRF2 and Rap1 binding to telomeric DNA R2 and R5 monitored by EMSA. Reaction mixtures contained the same amount of DNA (0.1 pmol). DNA duplexes were labelled with Alexa Fluor 488. A, C, E: show binding to telomeric duplex R2. B, D, F show binding to telomeric duplex R5. Gels in the first row (A, B) show the effect of protein Rap1 on DNA binding of TRF2. The second and third rows (C, D, E, F) represent binding of individual proteins to DNA substrates. The numbers above electrophoretic lanes represent molar ratios of proteins and DNA in individual wells. The reaction mixtures (15 µl) were resolved on horizontal 4% non-denaturing polyacrylamide gels.

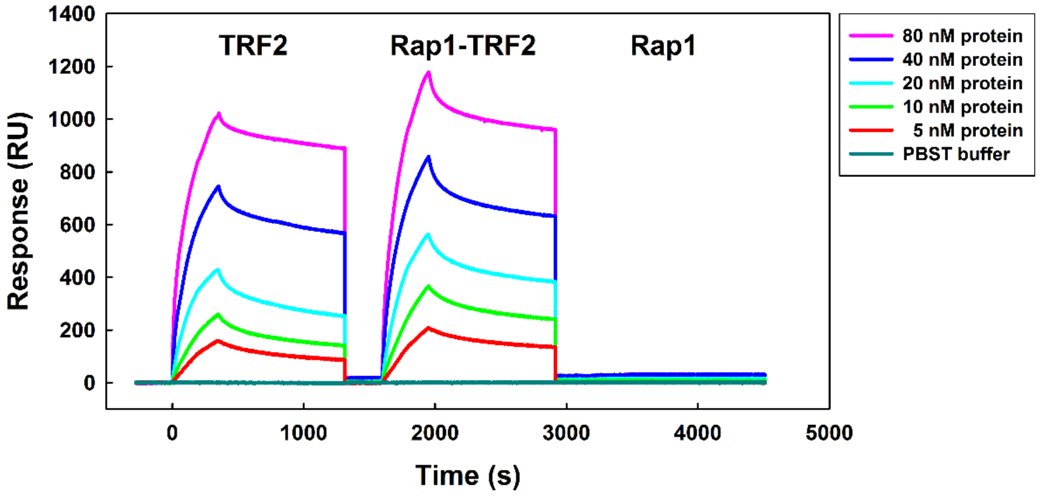

Figure S8. SPR sensorgrams of protein TRF2, complex Rap1-TRF2 and protein Rap1 binding to oligonucleotide SR2. The conditions of chip preparation and SPR analysis are described in Supplementary Materials and Methods. RU denominates response units.

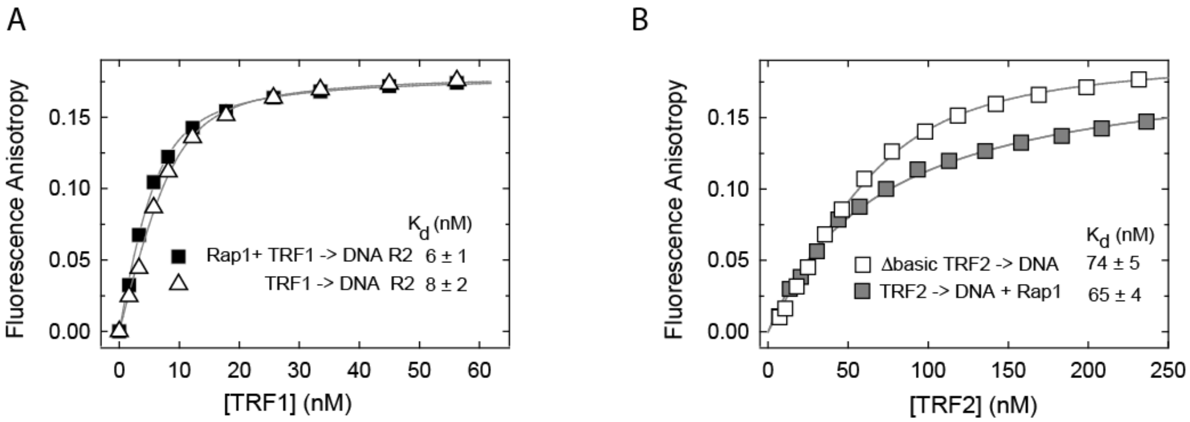

Figure S9. Rap1 does not affect TRF1 binding to telomeric DNA. (A) TRF1 was allowed to bind DNA duplex R2 (Alexa Fluor 488 labelled) with or without the presence of Rap1. The binding extent was monitored by fluorescence anisotropy. Note that Rap1 presence has no significant effect on dissociation constant for binding of TRF1 on DNA. **DNA binding affinity of TRF2 lacking N-terminal basic domain is similar to DNA binding affinity of TRF2 in presence of Rap1.** (B) Truncated version of TRF2 without N-terminal basic domain (Δbasic TRF2) was allowed to bind DNA duplex R2 (Alexa Fluor 488 labelled). The binding curve is compared with the binding curve for full-length TRF2 in presence of Rap1. The binding extent was monitored by fluorescence anisotropy. Note that Rap1 presence has similar effect on the full-length TRF2 dissociation constant as the removal of positively charged basic domain.

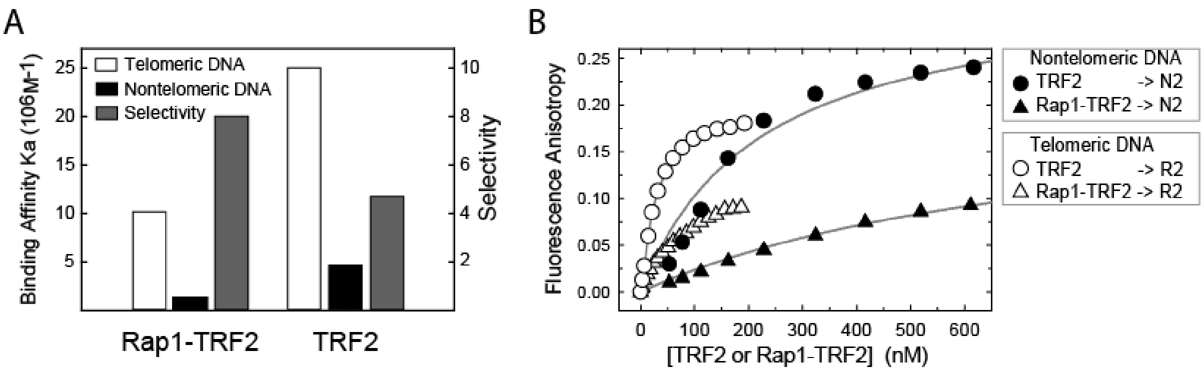

Figure S10. Rap1 improves selectivity of TRF2 binding to telomeric DNA. A: The quantification of binding association constant of TRF2 with or without of Rap1 to the telomeric DNA duplex R2 or nontelomeric DNA duplex N2 based on fluorescence anisotropy measurements (B). The comparison of binding affinity ratios for binding to telomeric and nontelomeric DNA (selectivity) revealed that Rap1 increases selective binding of TRF2 to telomeric DNA. Oligonucleotides in the cuvette (7.5 nM) were labelled by Alexa Fluor® 488. Measurement conditions were the same as described in materials and methods above. Note that binding saturations occur at significantly higher protein concentrations in case of protein binding to the nontelomeric DNA duplex N2 when compared to binding to the telomeric duplex R2.

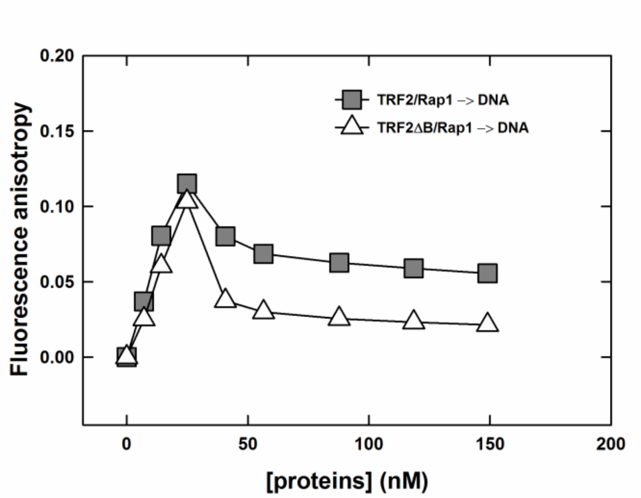

Figure S11. The release of telomeric DNA pre-bound to TRF2 full length or TRF2 lacking N-terminal basic domain (TRF2ΔB) after Rap1 addition. Either TRF2 or TRF2ΔB was allowed to bind with telomeric DNA R2 (7.5 nM) labelled by Alexa Fluor 488. The formation of TRF2-DNA or TRF2ΔB-DNA complex was demonstrated by an increase of anisotropy value. Next, instead of TRF2 or TRF2ΔB, Rap1 was added to the solution, which led to an immediate drop in the anisotropy. More DNA is released when TRF2ΔB is used, which is in accordance with our previous findings.

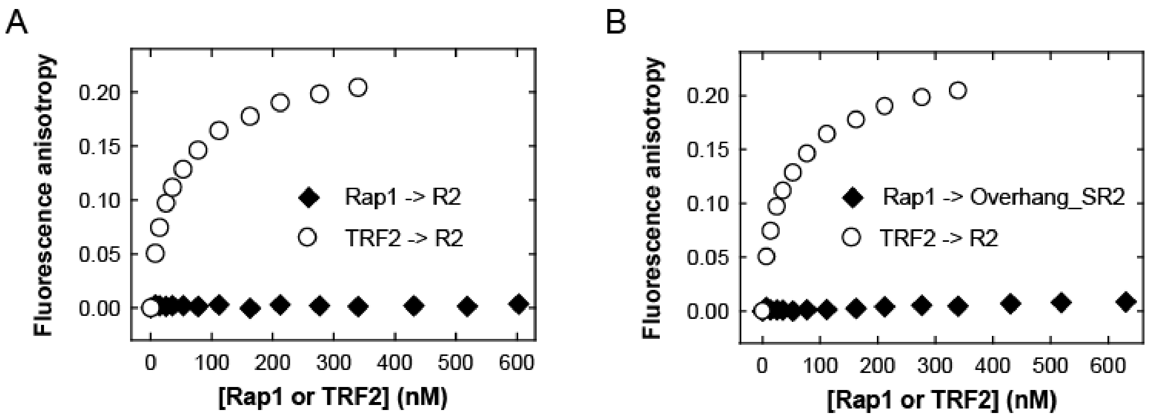

Figure S12. Rap1 does not bind DNA duplex R2 or Overhang_SR2. The interaction of Rap1 to the fully hybridized DNA duplex R2 (A) or Overhang_SR2 (B) was monitored by fluorescence anisotropy and compared with TRF2 binding to DNA duplex R2. Oligonucleotides in the cuvette (7.5 nM) were labelled by Alexa Fluor® 488. Assay conditions were the same as described in materials and methods above. Note that binding was not observed even at significantly higher Rap1 concentrations compared to TRF2 concentrations.

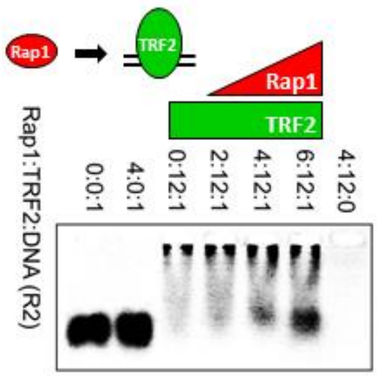

Figure S13. Rap1 induces a partial release of TRF2 from a preformed TRF2-DNA complex also at DNA concentrations well below Kd. Reaction mixtures contained the same amount of DNA (0.1 pmol), constant amount of TRF2 (1.2 pmol) and increasing amount of Rap1 (0.2-0.4 pmol). The numbers above electrophoretic lanes represent molar ratios of proteins and DNA in individual wells. Note that the saturation molar ratio of TRF2-DNA complex had to be changed due to the DNA concentration being significantly lower than Kd of TRF2 binding to DNA. The binding saturation occurs at TRF2:DNA ratio 12:1. DNA duplex R2 was labelled with Alexa Fluor 488. The reaction mixtures (15 μl) were resolved on horizontal 4% non-denaturing polyacrylamide gel.

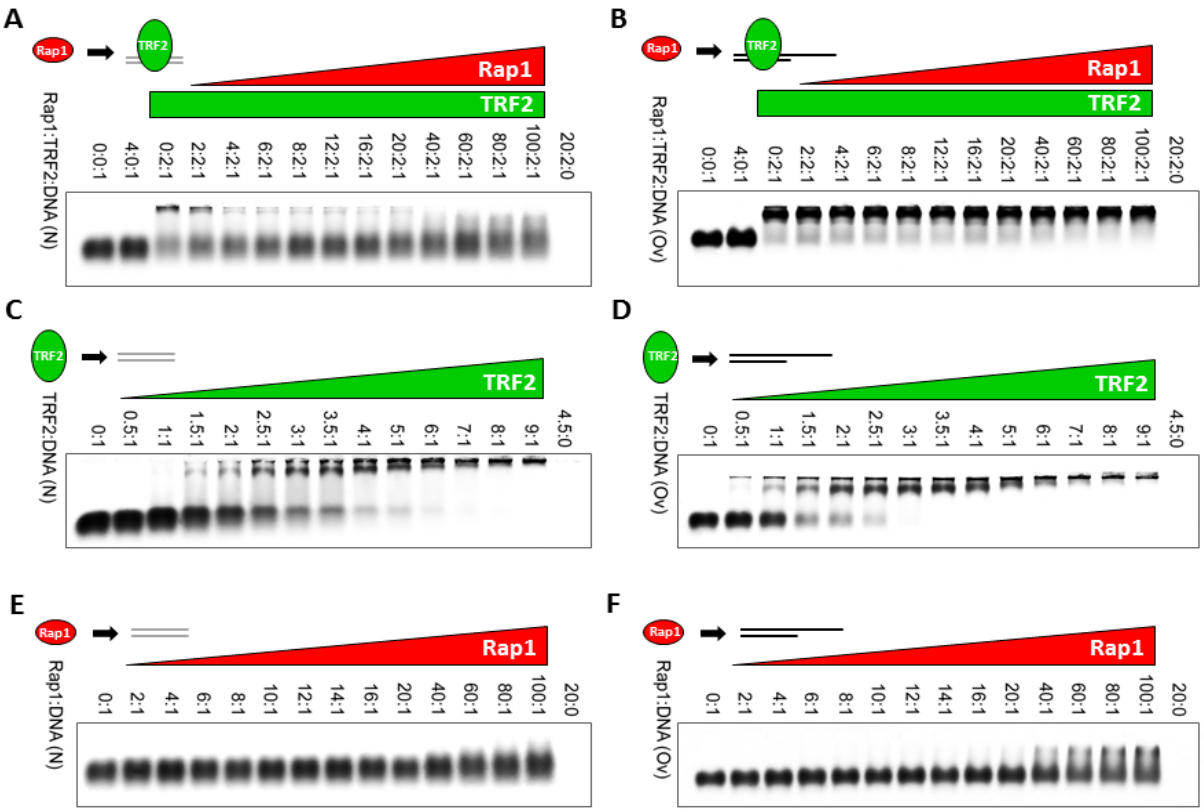

Figure S14. TRF2 and Rap1 binding to non-telomeric N and overhang Ov DNA monitored by EMSA. Reaction mixtures (15 µl) contained the same amount of DNA (3 pmol). DNA duplexes were labelled with fluorophore Alexa Fluor 488. A, C, E show protein binding to non-telomeric duplex N. B, D, F: show protein binding to DNA overhang Ov. Gels in the first row (A, B) show the effect of protein Rap1 on DNA binding of TRF2. The second and third rows (C, D, E, F) describe binding of individual proteins to DNA. The numbers above electrophoretic lanes represent the molar ratios of proteins and DNA in individual wells. The reaction mixtures were resolved on horizontal 4% non-denaturing polyacrylamide gels. Note that after addition of Rap1 to TRF2 pre-bound to DNA Ov with single-stranded overhang (B) no release of free DNA is observed.
